# Supplementary material for: Multi-electron nitrobenzothiadiazole sp-conjugated-alkynyl covalent organic frameworks for ammonium-ion batteries
Source: Nat Commun. 2026 Mar 7;17:3599. doi: 10.1038/s41467-026-70370-x (PMC13096558; doi:10.1038/s41467-026-70370-x)
Supplement: Supplementary file 1 — Supplementary Information [file 41467_2026_70370_MOESM1_ESM.pdf]

Supplementary Information

Multi-Electron Nitrobenzothiadiazole *sp*-Conjugated-Alkynyl Covalent Organic Frameworks for Ammonium-Ion Batteries

Yumin Chen[1], Da Zhang[1], Yang Qin[1], Chengmin Hu[2], Ling Miao[1], Yaokang Lv[3], Ziyang Song[1,4]*, Lihua Gan[1,5]*, Mingxian Liu[1,5]*

[1]Shanghai Key Lab of Chemical Assessment and Sustainability, School of Chemical Science and Engineering, Tongji University, 1239 Siping Rd., Shanghai, 200092, P. R. China.

[2]Department of Chemistry, State Key Laboratory of Molecular Engineering of Polymers, Laboratory of Advanced Materials, Shanghai Key Lab of Molecular Catalysis and Innovative Materials, Fudan University, 2005 Songhu Rd., Shanghai, 200433, P. R. China.

[3]College of Chemical Engineering, Zhejiang University of Technology, 18 Chaowang Rd., Hangzhou, 310014, P. R. China.

[4]State Key Laboratory of Pollution Control and Resource Reuse, College of Environmental Science and Engineering, Advanced Research Institute, Tongji University, 1239 Siping Rd., Shanghai, 200092, P. R. China.

[5]State Key Laboratory of Cardiovascular Diseases and Medical Innovation Center, Shanghai East Hospital, School of Medicine, Tongji University, 150 Jimo Rd., Shanghai, 200120, P. R. China.

*Correspondence: songziyang@tongji.edu.cn (Z. Song); ganlh@tongji.edu.cn (L. Gan); liumx@tongji.edu.cn (M. Liu)

Section S1. Supplementing Characterizations

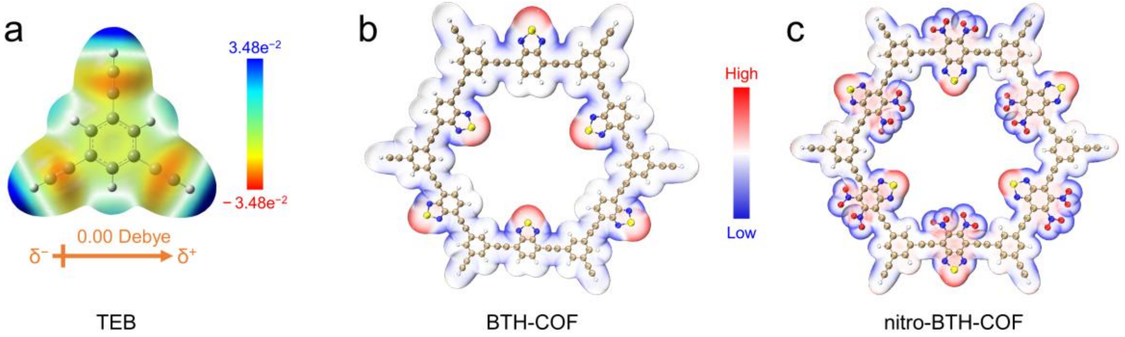

Supplementary Fig. 1 | Molecular ESP analysis. a TEB, **b** BTH-COF, **c** nitro-BTH COF. Colours of elements: H, white; C, brown; O, red; N, blue; S, yellow.

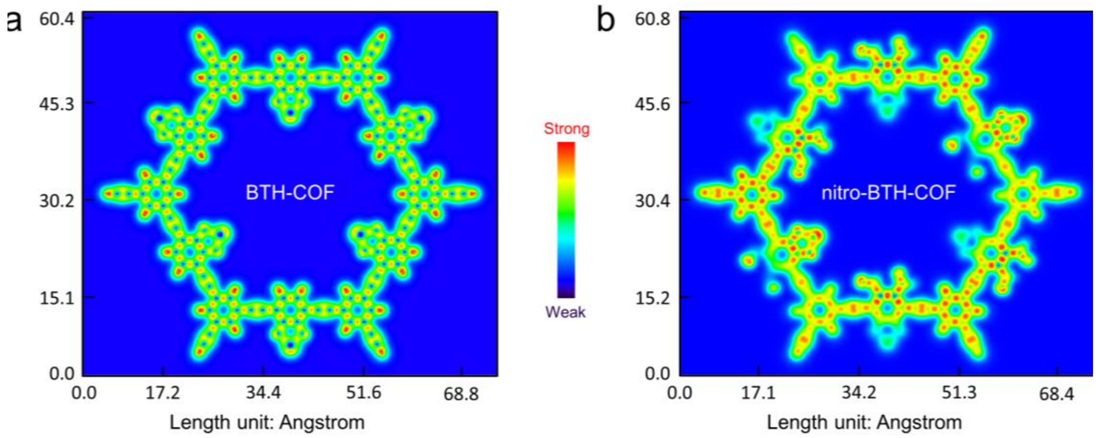

Supplementary Fig. 2 | Analysis of π-electron localization function. a BTH-COF and **b** nitro-BTH COF.

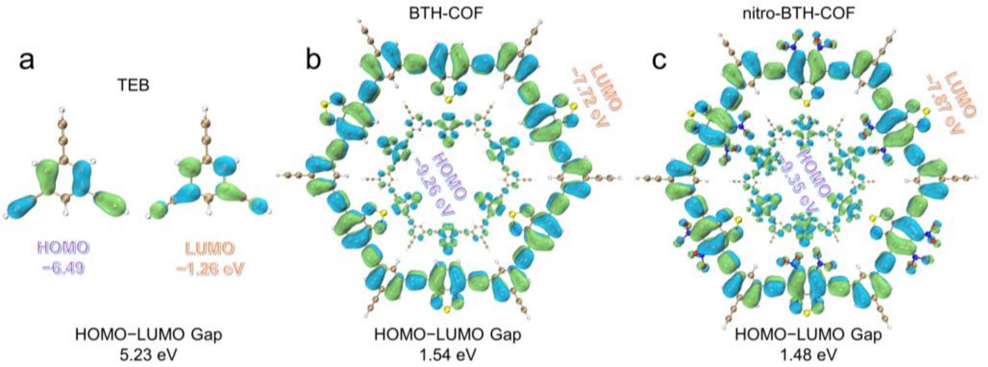

Supplementary Fig. 3 | Energy levels and frontier molecular orbitals. a TEB, **b** BTH-COF, **c** nitro-BTH COF. Colours of elements: H, white; C, brown; O, red; N, blue; S, yellow.

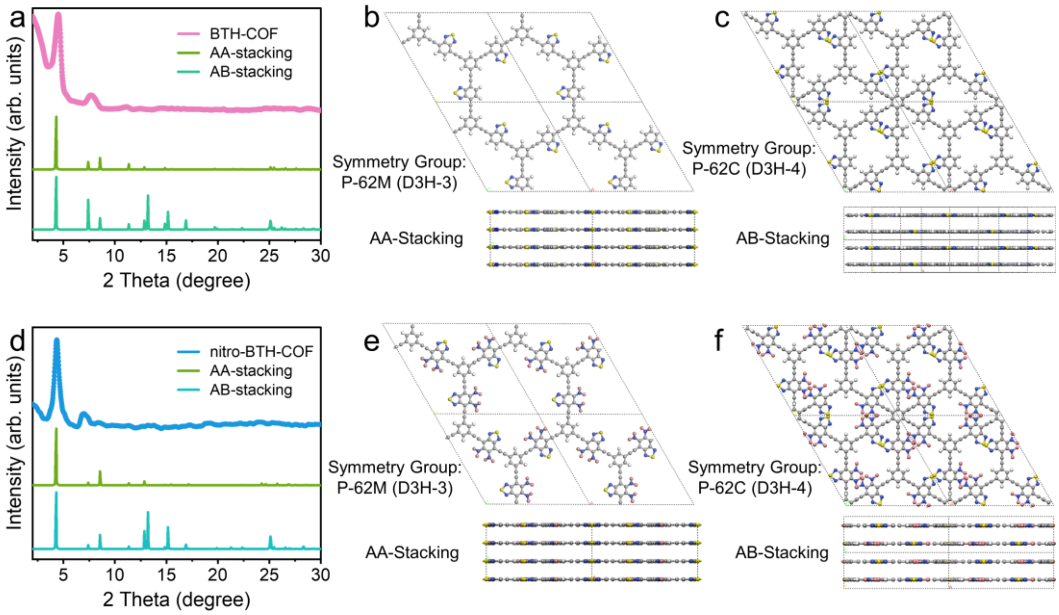

Supplementary Fig. 4 | Experimental PXRD patterns and simulated crystal structural models. **a−c** BTH-COF and **d−f** nitro-BTH-COF. Colours of elements: H, white; C, grey; O, red; N, blue; S, yellow.

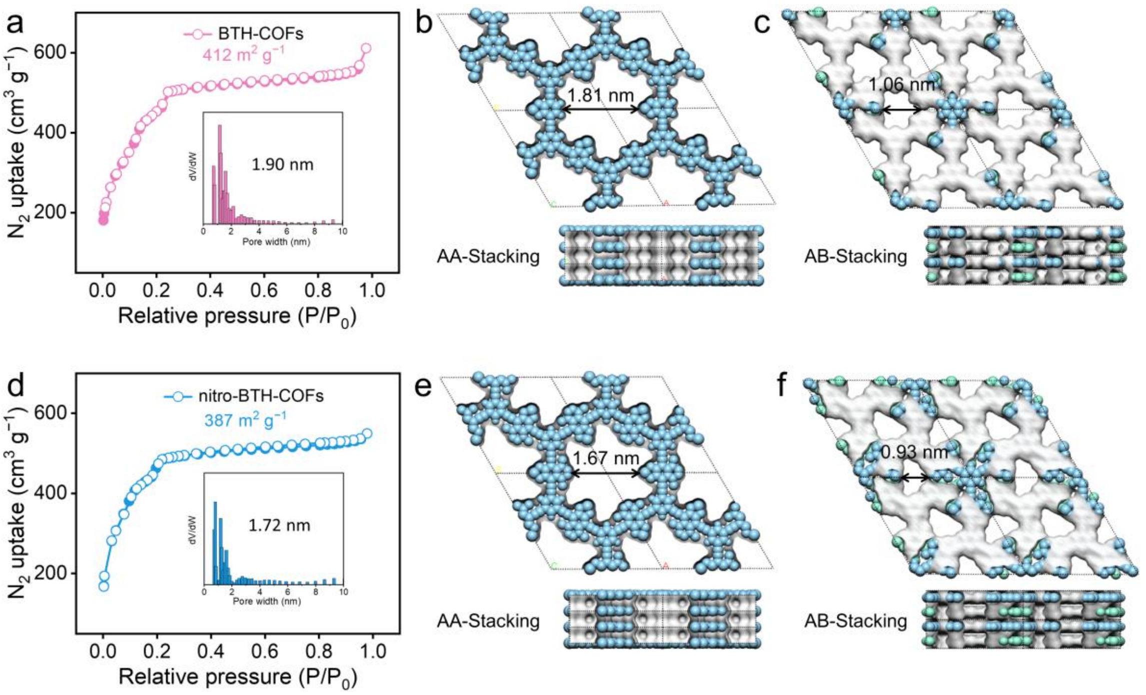

Supplementary Fig. 5 | Nitrogen adsorption/desorption isotherms and simulated pore-size distribution models. a−c BTH-COF and **d−f** nitro-BTH COF.

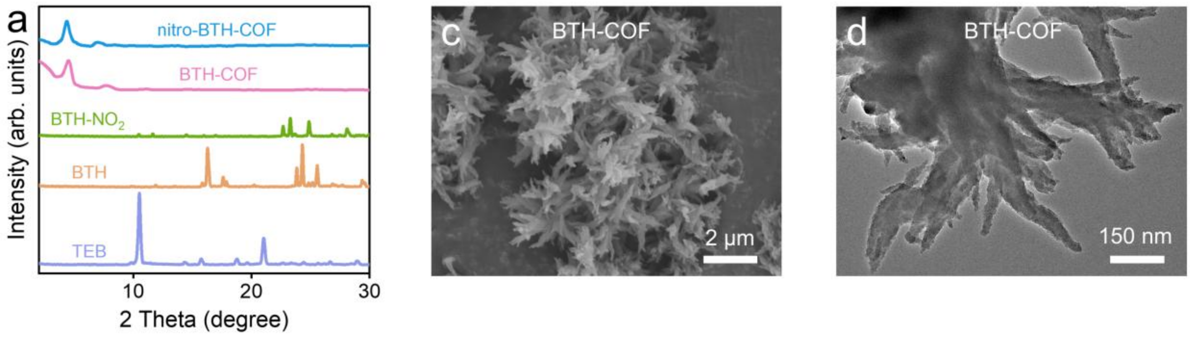

Supplementary Fig. 6 | Structural and morphological characterization. a Experimental PXRD patterns of monomers, BTH-COF, and nitro-BTH-COF. **b** SEM and **c** TEM images of BTH-COF.

Notes to Supplementary Fig. 6: XRD patterns of TEB, BTH, and BTH-NO_2 monomers show diffraction peaks at 10−30°, which completely disappear after Sonogashira coupling reaction, triggering the emergence of distinctive diffraction peaks at 3.5° for highly crystalline BTH-COF and nitro-BTH-COF.

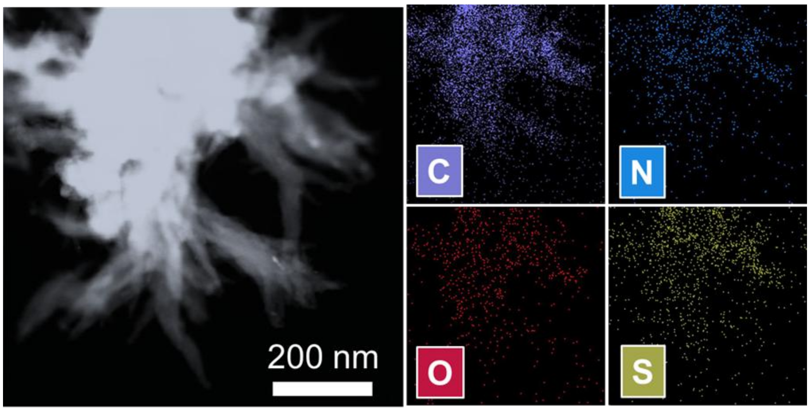

Supplementary Fig. 7 | Element distribution maps of nitro-BTH-COF.

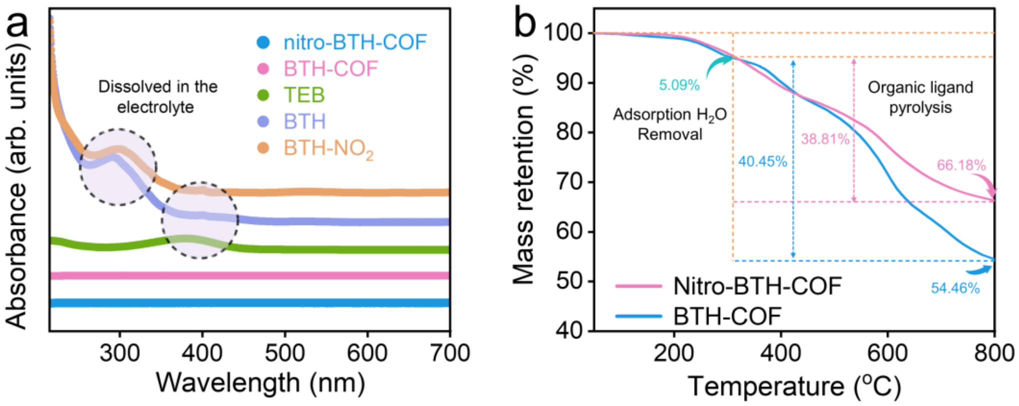

Supplementary Fig. 8 | Chemical and thermal stability analysis. a UV/Vis spectra of BTH-COF and nitro-BTH-COF soaked in 2 M $(NH_4)_2SO_4$ aqueous electrolyte. **b** Thermogravimetric analysis of BTH-COF and nitro-BTH-COF.

<u>**Notes to Supplementary Fig. 8:**</u> Compared with soluble small molecules of TEB, BTH and BTH-NO_2 (Supplementary Fig. 8a), there is no UV-vis absorption signal for both BTH-COF and nitro-BTH-COF after soaking 2 M $(NH_4)_2SO_4$ aqueous electrolyte, confirming their structural robustness and anti-dissolution ability. The rigid alkynyl-bridged *sp*-conjugated frameworks of both BTH-COF and nitro-BTH-COF with a unique pore structure contribute to their structural stability, which is beneficial for sustained electrochemical activity.

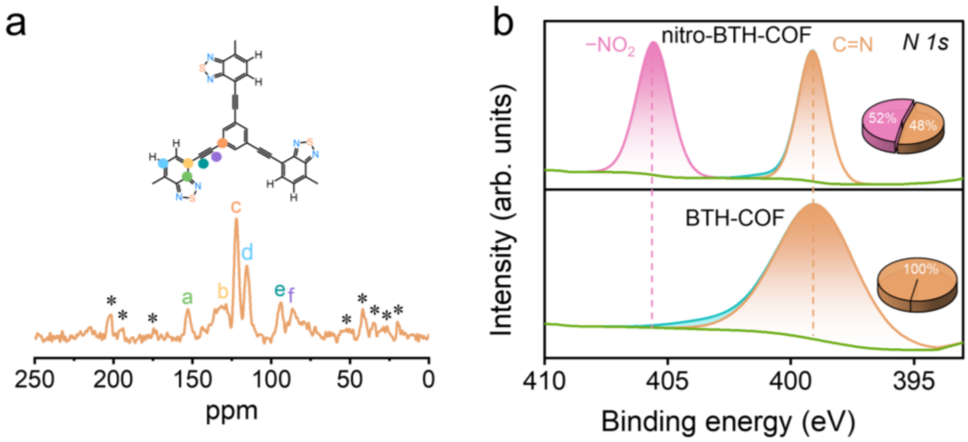

Supplementary Fig. 9 | Spectroscopic characterization. a Solid-state ^{13}C NMR spectrum of BTH-COF. **b** High-resolution *N 1s* XPS spectra of nitro-BTH-COF and BTH-COF.

Section S2. Supplementary Electrochemistry Analysis

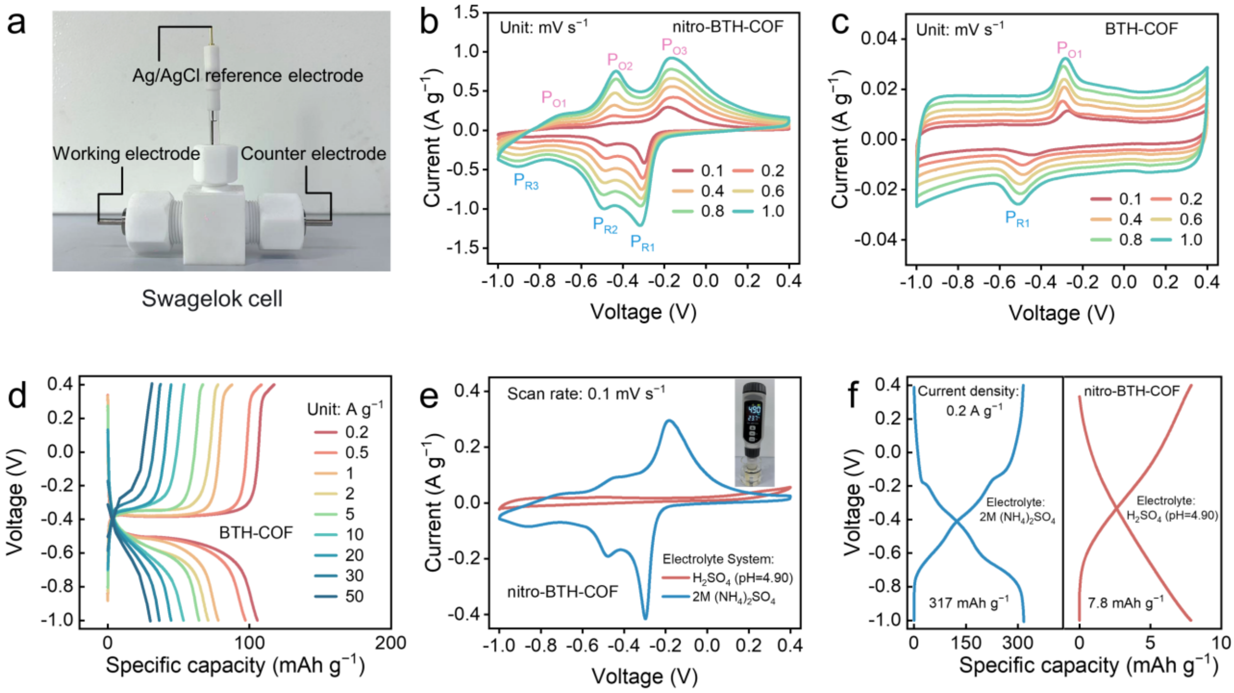

Supplementary Fig. 10 | Electrochemical performance characterization. a Optical image of Swagelok cell. **b** CV curves of nitro-BTH-COF at different scan rates. **c** CV curves of BTH-COF. **d** GCD profiles of BTH-COF at different (dis)charge current density. Electrochemical performance of nitro-BTH-COF at different electrolyte system: **e** CV curves at 0.1 mV s^{-1}, **f** GCD profiles at 0.2 A g^{-1}.

Notes to Supplementary Fig. 10: nitro-BTH-COF was served as the negative electrode (rather than the positive electrode) in AIBs. Electron-withdrawing nitro groups act as electron acceptors to easily undergo reduction reaction to couple NH_4^+ ions, which are dependent of the LUMO energy level. Indeed, the introduction of nitro groups lowers the LUMO energy level from −7.72 (BTH-COF) to −7.87 eV (nitro-BTH-COF, Supplementary Fig. 3), resulting in a slight positive shift in the average redox potential from −0.56 V (BTH-COF, Supplementary Fig. 10d) to −0.49 V (nitro-BTH-COF, Fig. 2b). Of note, with the ability to accept two electrons per nitro group, it can trigger extra multielectron redox reactions for nitro-BTH-COF negative electrode to achieve a superior capacity of 317 mAh g^{-1} compared to BTH-COF negative electrode (117 mAh g^{-1}, Supplementary Fig. 10d). Overall, nitro groups of high-capacity nitro-BTH-COF negative electrode do not significantly damage the voltage (1.1 V) of nitro-BTH-COF||NiFeHCF full battery, which thus

liberates state-of-the-art battery-level specific energy (86.1 Wh $kg^{-1}{}_{cell}$) among all reported NH_4^+ full batteries (Fig. 5f and Supplementary Table 2).

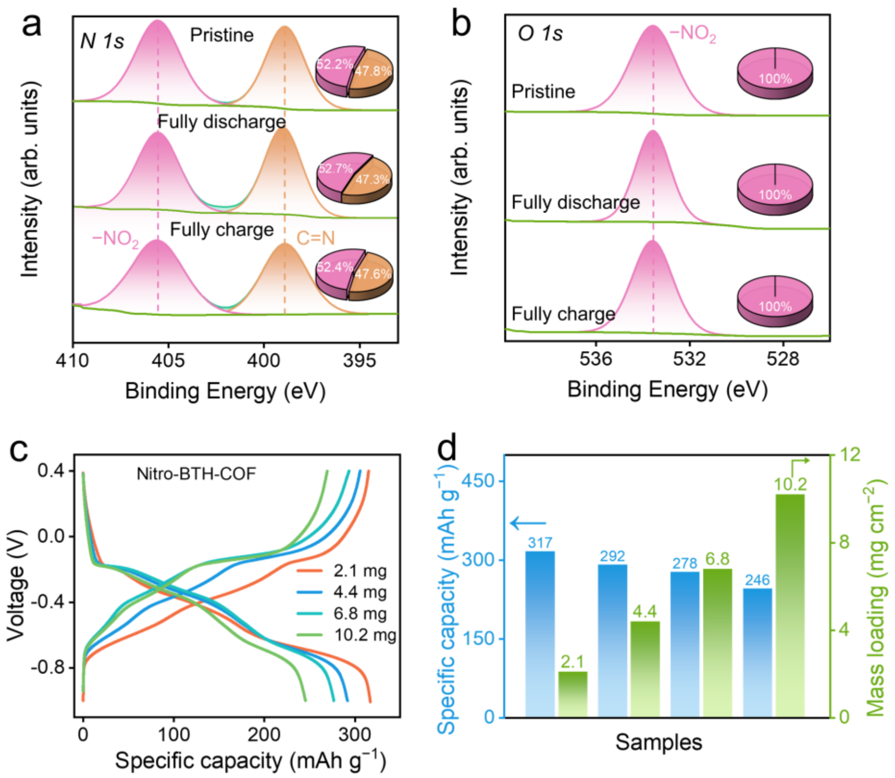

Supplementary Fig. 11 | Mechanism analysis and loading-dependent performance. High-resolution **a** *N 1s* and **b** *O 1s* XPS spectra of nitro-BTH-COF negative electrode in H_2SO_4/H_2O electrolyte (pH=4.90) during the electrochemical process. **c** GCD profiles and **d** capacities of nitro-BTH-COF negative electrode at different mass loadings.

Notes to Supplementary Figs. 10 and 11: Considering the weak acidity of 2 M $(NH_4)_2SO_4/H_2O$ electrolyte (pH=4.90) and the small size of H^+ ions, the contribution of H^+ ion to the total capacity storage of nitro-BTH-COF negative electrode was studied in H_2SO_4/H_2O electrolyte (with the same pH value as 2 M $(NH_4)_2SO_4/H_2O$ electrolyte). As previously reported[S11], H^+ co-intercalation is a well-documented phenomenon in AIBs. In our case, nitro-BTH-COF negative electrode in H_2SO_4/H_2O electrolyte exhibits completely different redox peaks and charge storage behaviors (Supplementary Fig. 10e) with a negligible capacity of 7.8 mAh g^{-1} (Supplementary Fig. 10f), far below the 317 mAh g^{-1} observed in $(NH_4)_2SO_4/H_2O$ electrolyte. This result excludes H^+ involvement in the redox process of nitro-BTH-COF negative electrode. Furthermore, we investigated the high-resolution *N 1s* and *O 1s* XPS spectra of nitro-BTH-COF negative electrode in H_2SO_4/H_2O electrolyte at different electrochemical states. During the electrochemical process, the C=N (398.9 eV) and $-NO_2$ groups (405.6/533.5 eV) of nitro-BTH-COF negative electrode remain unchanged without significant shifts or impure signal formation caused by H^+ reaction

(Supplementary Fig. 11a and b). These results confirm that the role of H^+ ions can be ignored, and NH_4^+ ions are primarily responsible for the capacity storage of nitro-BTH-COF negative electrode.

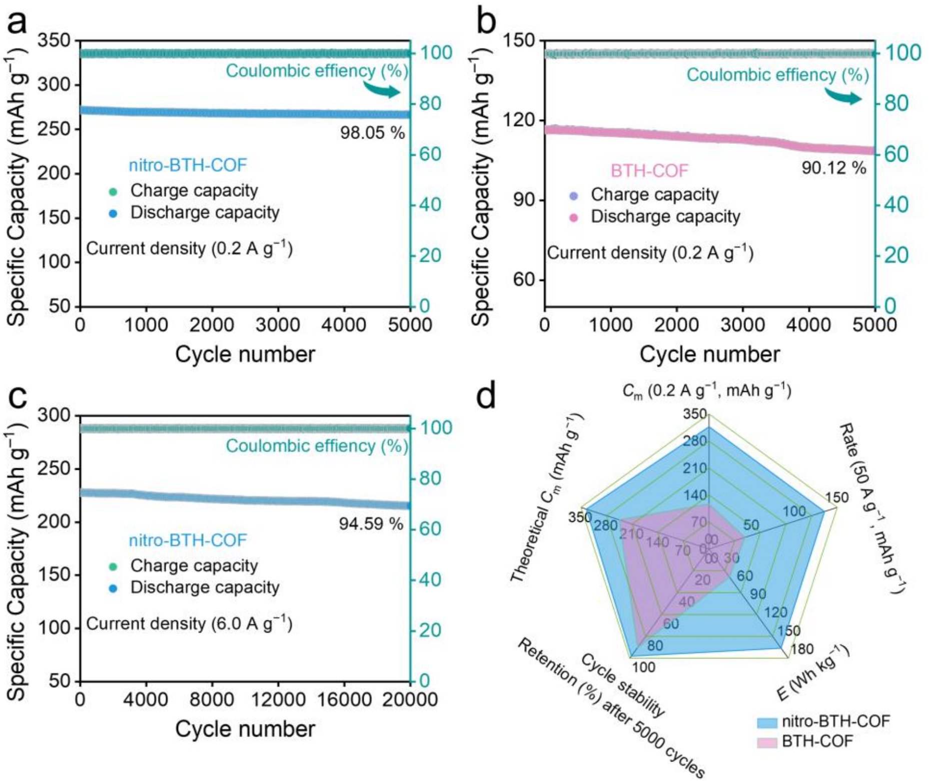

Supplementary Fig. 12 | Cycling stability and performance comparison. Cycling performance of **a** nitro-BTH-COF and **b** BTH-COF negative electrodes at 0.2 A g^{-1}. **c** Cycling performance of nitro-BTH-COF negative electrode at 6 A g^{-1}. **d** Radar diagram of overall comparison of electrochemical performance between nitro-BTH-COF and BTH-COF.

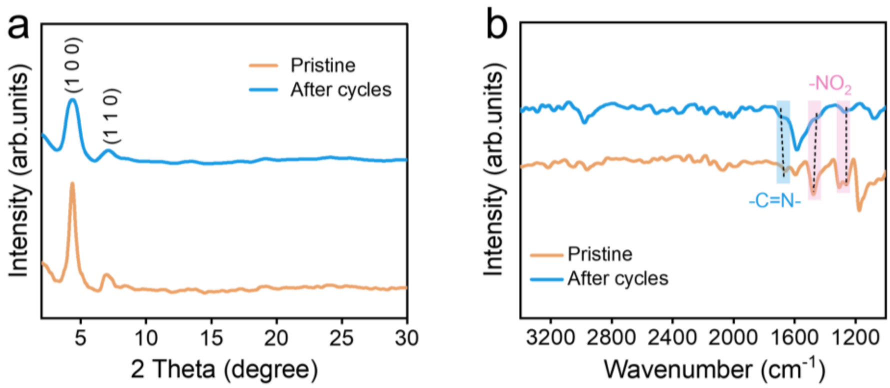

Supplementary Fig. 13 | Structural characterizations of nitro-BTH-COF negative electrode before and after cycles. **a** XRD patterns and **b** FT-IR spectra.

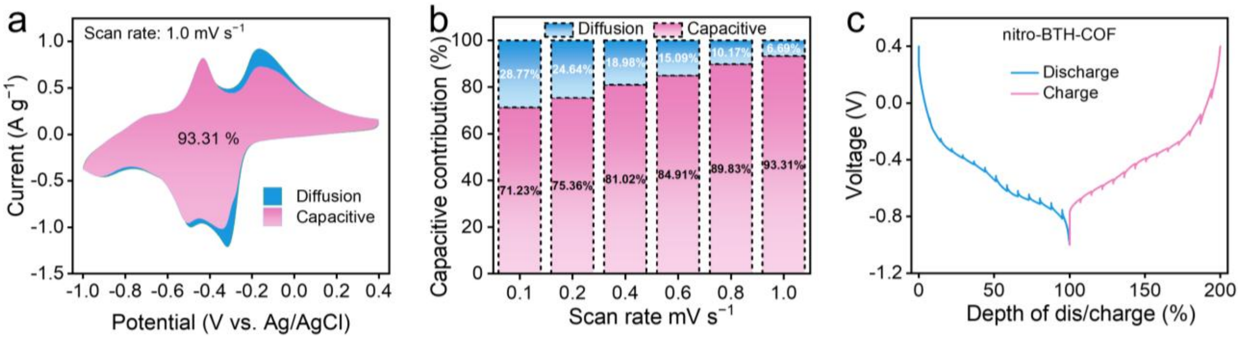

Supplementary Fig. 14 | Charge storage kinetics of nitro-BTH-COF negative electrode. a Capacitive contribution. **b** Ratios of capacitive and diffusion-controlled contributions at various scan rates. **c** GITT curves.

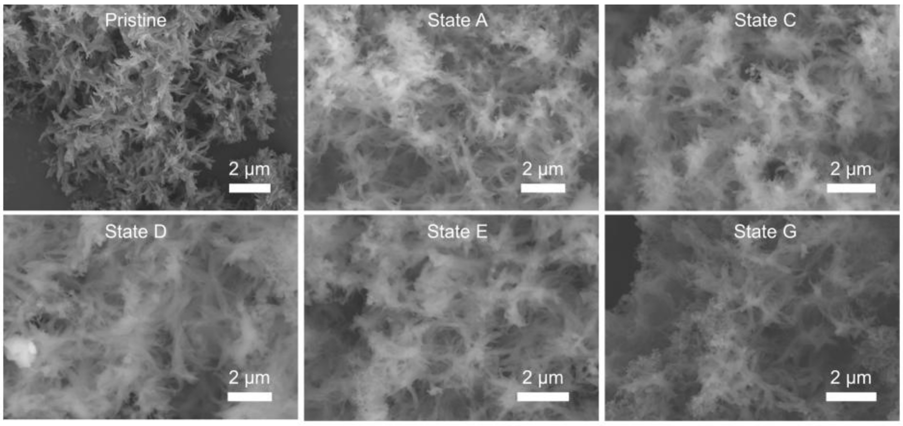

Supplementary Fig. 15 | SEM images of nitro-BTH COF negative electrode at different discharge/charge states. The batteries were first pre-cycled at a specific current of 1 A g^{-1} for 3 cycles at 25 ± 0.5 °C under ambient pressure. Subsequently, the cycling was interrupted at specific depth of (dis)charge states to allow for disassembly and ex-situ characterization.

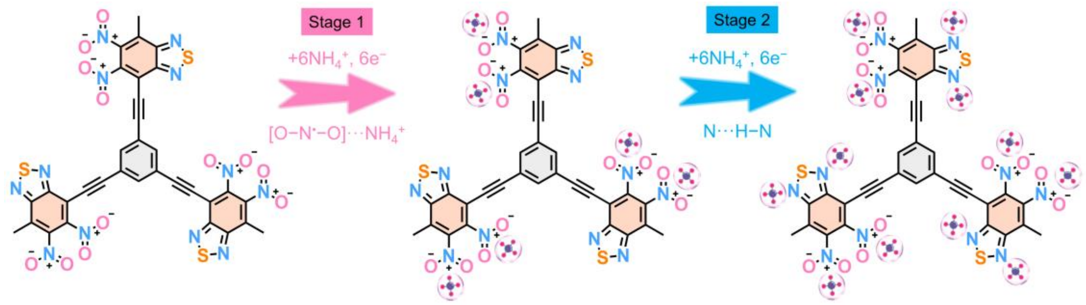

Supplementary Fig. 16 | Two-stage NH_4^+ storage mechanism of nitro-BTH COF negative electrode.

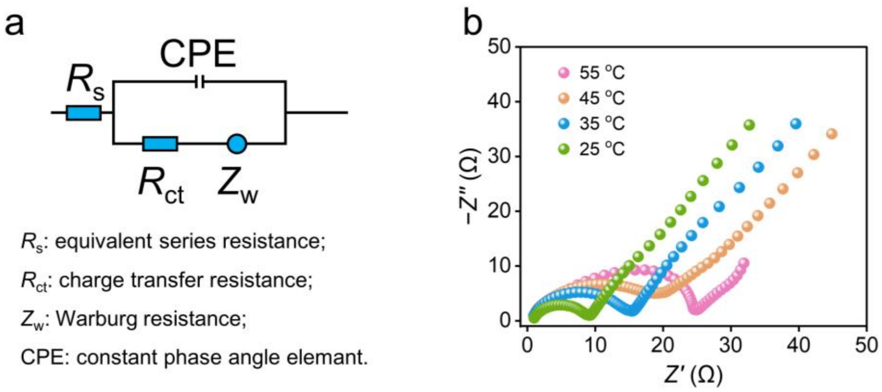

Supplementary Fig. 17 | Electrochemical kinetic analysis. a Equivalent circuit of Nyquist plots. **b** Electrochemical impedance spectra (EIS) of nitro-BTH COF negative electrode at different operation temperatures for calculating the activation energy.

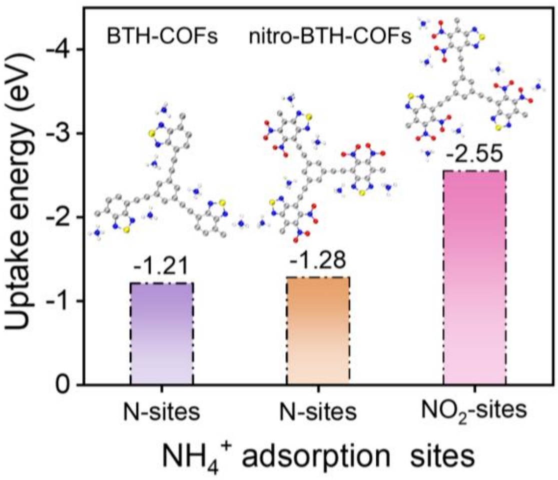

Supplementary Fig. 18 | Theoretical calculation of uptake energetics. Geometrically optimized molecular plane and corresponding uptake energy at NO_2/C=N active sites of BTH-COF and nitro-BTH-COF. Colours of elements: H, white; C, grey; O, red; N, blue; S, yellow.

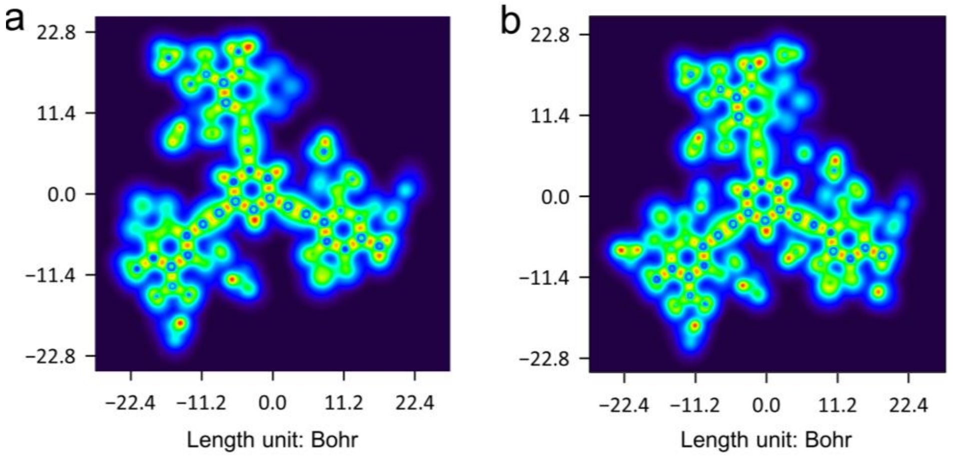

Supplementary Fig. 19 | Electronic structure evolution. LOL-π maps of **a** state I (nitro-BTH-COF-6NH_4^+) and **b** state II (nitro-BTH-COF-12NH_4^+).

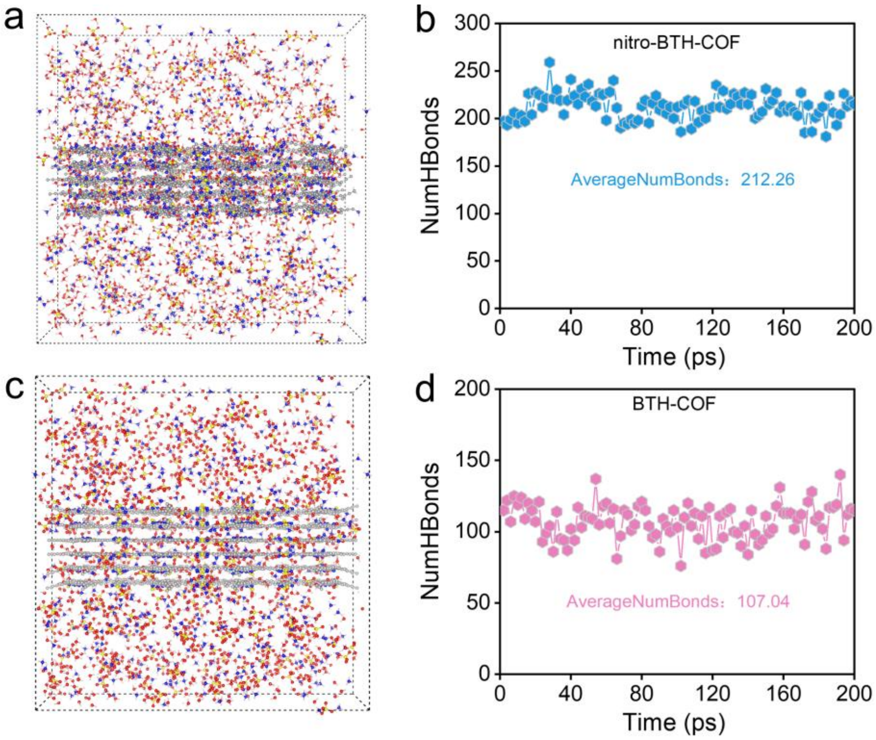

Supplementary Fig. 20 | MD simulations of interfacial interactions. The snapshots of **a** nitro-BTH-COF in 2M $(NH_4)_2SO_4$ electrolyte **c** BTH-COF in 2 M $(NH_4)_2SO_4$ electrolyte. H-bonding number between C=N/NO_2 active sites and NH_4^+ ions. **b** nitro-BTH-COF and **d** BTH-COF. Colours of elements: H, white; C, grey; O, red; N, blue; S, yellow.

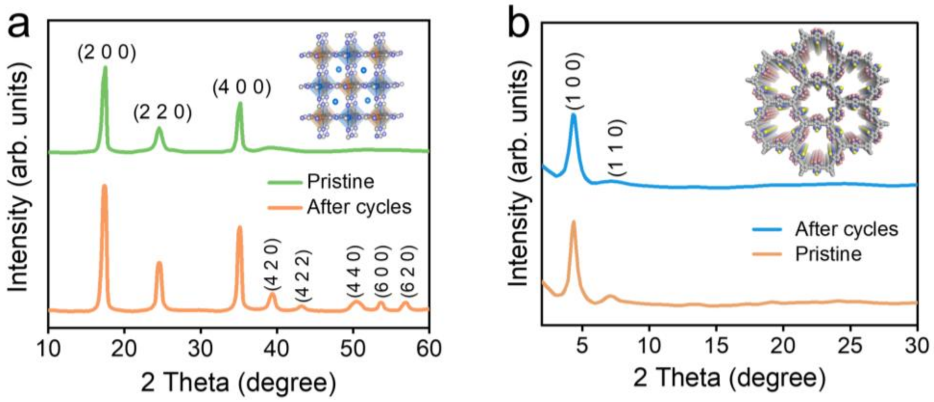

Supplementary Fig. 21 | Structural stability verification. XRD patterns of **a** NiFeHCF positive electrode and **b** nitro-BTH-COF negative electrode before and after cycles.

Supplementary Table 1 | Comparison of capacity and cycling life of recently reported organic electrode materials for AIBs in the literatures.

Materials	Electrode mass loading	C_m (mAh g^{-1})	Life	Refs.
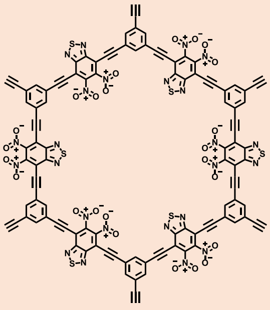 nitro-BTH-COF	2.1 mg cm^{-2}	317@0.2 A g^{-1}	93.7%, 70,000 cycles, 20 A g^{-1} and 94.6%, 20,000 cycles, 6 A g^{-1}	This work
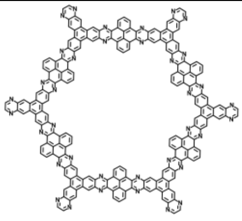 Aza-based COF (HATP-PT COF)	5.0 mg cm^{-2}	109@0.5 A g^{-1}	89%, 20,000 cycles, 6 A g^{-1}	[S1]
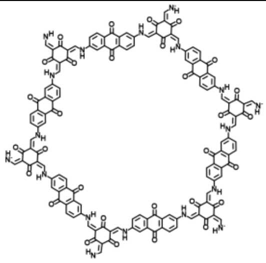 Anthraquinone-based COF (DAAQ-TP-COF)	N/A	141@0.1 A g^{-1}	90%, 8000 cycles, 6 A g^{-1}	[S2]
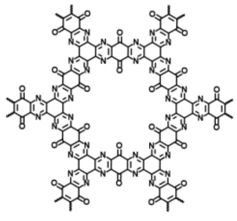 Quinone/pyrazine COF (QA-COF)	5.0 mg cm^{-2}	220@0.5 A g^{-1}	~80%, 7000 cycles 6 A g^{-1}	[S3]
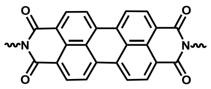 Polyimide (PTCDI)	0.5–1.0 mg cm^{-2}	130@0.12 A g^{-1}	64%, 5000 cycles 10 A g^{-1}	[S4]

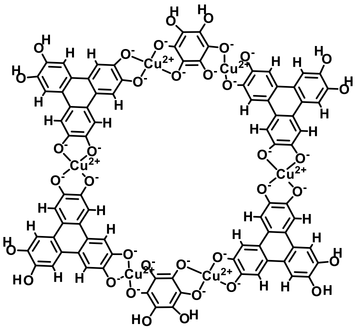<br>(Cu-HHTP-THBQ)	1.2-1.5 mg cm^{-2}.	265@0.2 A g^{-1}	88.2%, 12,000 cycles<br>5 A g^{-1}	[S5]
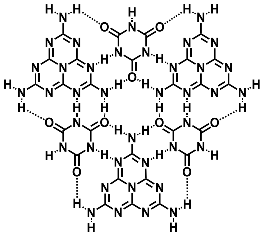<br>Organic Superstructures<br>(OSs)	3.0 mg cm^{-2}	393@0.2 A g^{-1}	85.8%, 60,000 cycles,<br>10 A g^{-1}	[S6]
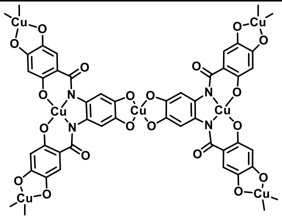<br>(Cu-HHB-MOF)	N/A	111.7 mF cm^{-2}<br>@0.4 mA cm^{-2}	85.8%, 10,000 cycles,<br>2 mA cm^{-2}	[S7]
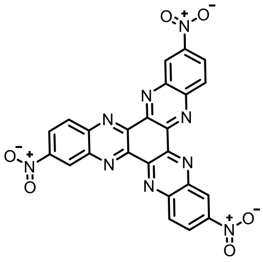<br>Trinitrodiquinoxalino<br>phenazine (HATNTN)	1.5 mg cm^{-2}	246.4@1 A g^{-1}	88.1%, 30,000 cycles,<br>3 A g^{-1}	[S8]

Supplementary Table 2 | Fitting values of EIS equivalent circuit of negative electrode at different temperatures.

Temperature	BTH-COF			nitro-BTH-COF		
(°C)	R_s (Ω)	R_{ct} (Ω)	X^2	R_s (Ω)	R_{ct} (Ω)	X^2
25	1.37	40.4	2.1×10^{-6}	1.08	28.1	6.3×10^{-7}
35	1.30	38.2	0.4×10^{-9}	0.97	22.7	1.6×10^{-6}
45	1.17	31.5	3.6×10^{-7}	0.94	18.2	0.2×10^{-9}
55	1.01	20.4	0.8×10^{-8}	0.51	9.8	3.2×10^{-9}

Note: R_s denotes the equivalent series resistance, R_{ct} represents the charge transfer resistance, and X^2 indicates the fitting error.

Supplementary Table 3 | Comparison of battery-level specific energy (*E*, Wh kg^{-1}) and cycling life of recently reported AIBs in the literatures.

Negative electrode (Mass loading)	Positive electrode (Mass loading)	*E* (N/P ratio)	Lifespan	Refs.
nitro-BTH-COF (2.2 mg cm^{-2})	NiFeHCF (5.2 mg cm^{-2})	86.1 (1.12)	88.91%, 25,000 cycles, 10 A g^{-1}	This Work
HATP-PT COF (5.0 mg cm^{-2})	CuHCF (5.0 mg cm^{-2})	30 (0.45)	89%, 20,000 cycles, 1 A g^{-1}	[S1]
DAAQ-TP-COF (N/A)	PANI (N/A)	N/A (~1.1)	81%, 1000 cycles, 6 A g^{-1}	[S2]
OSs (3.0 mg cm^{-2})	OSs-R (3.0 mg cm^{-2})	81 (~1.0)	90.3%, 100,000 cycles, 5 A g^{-1}	[S6]
PTCDI (0.5 mg cm^{-2})	CuHCF (0.5 mg cm^{-2})	63.1 (~1.0)	82.8%, 500 cycles, 1 A g^{-1}	[S9]
PTCDI (0.6 mg cm^{-2})	N-CuHCF (1.2 mg cm^{-2})	31 (0.94)	72%, 1000 cycles, 10 C	[S10]
PTCDI (N/A)	$VOPO_4 \cdot 2\,H_2O$ (3.0 mg cm^{-2})	N/A (1.2)	99.6%, 500 cycles, 0.1 A g^{-1}	[S11]
PNNI (3.5 mg cm^{-2})	Ni-APW (N/A)	68.7 (~1.0)	100%, 10,000 cycles, 1 A g^{-1}	[S12]
PTCDI (N/A)	MnHCF (2.98 mg cm^{-2})	N/A (~1.0)	~71.1%, 70 cycles, 0.03 A g^{-1}	[S13]
PTCDI (4.5 mg cm^{-2})	MnAl-LDH (1.5 mg cm^{-2})	45.8 (0.94)	92%, 100 cycles, 0.1 A g^{-1}	[S14]
PANI N/A	$NH_4V_4O_{10}$ N/A	N/A (N/A)	73.3%, 1000 cycles, 0.1 A g^{-1}	[S15]
PTCDI (4.0–5.0 mg cm^{-2})	A-PBA N/A	55.5 (1.2)	72.3%, 4000 cycles, 0.4 A g^{-1}	[S16]
PTCDI (2.0–5.0 mg cm^{-2})	Ni-APW (2.0–5.0 mg cm^{-2})	43 (0.5)	67%, 1000 cycles, 0.12 A g^{-1}	[S17]
PTCDI@MXene (5.0 mg cm^{-2})	CuHCF (5.0 mg cm^{-2})	41.5 (1.2)	77%, 2000 cycles, 0.5 A g^{-1}	[S18]
PTCDI (2.0–3.0 mg cm^{-2})	FeMnHCF (1.5–2.5 mg cm^{-2})	71 (1.4)	67%, 3000 cycles, 9 A g^{-1}	[S19]

PTCDI<br>(1.5–2.0 mg cm^{-2})	A-MnPBA<br>(1.5–2.0 mg cm^{-2})	65<br>(1.2)	73.9%, 10,000 cycles, 10 A g^{-1}	[S20]
PTCDI<br>(0.7 mg cm^{-2})	MnO_x/MnS_2<br>(1.0 mg cm^{-2})	79.6<br>(N/A)	90%, 15,000 cycles, 20 A g^{-1}	[S21]
QDAQ<br>(4.0 mg cm^{-2})	QDAQ-R<br>(4.0 mg cm^{-2})	N/A<br>(N/A)	90%, 15,000 cycles, 20 A g^{-1}	[S22]
M_oS_2@PANI<br>(N/A)	M_oS_2@PANI<br>(N/A)	59.8<br>(N/A)	80.5%, 10,000 cycles, 10 A g^{-1}	[S23]
pEP(NQ)E<br>(10 mg cm^{-2})	pEP(QH2)E<br>(10 mg cm^{-2})	N/A<br>(~1.0)	85%, 500 cycles, 0.2 A g^{-1}	[S24]
PUQ<br>(N/A)	$PTC(QH_2)$<br>(1.5 mg cm^{-2})	56.2<br>(1.05)	80%, 1000 cycles, 2 A g^{-1}	[S25]
Poly-NAPD<br>(2.0 mg cm^{-2})	NiHCF<br>(7.0 mg cm^{-2})	31.8<br>(0.91)	85.5%, 500 cycles, 1.0 A g^{-1}	[S26]
PANI<br>(N/A)	$NH_4V_3O_8 \cdot 2.9H_2O$<br>(N/A)	16.5<br>(N/A)	95%, 400 cycles, 0.1 A g^{-1}	[S26]
PANI<br>(5.0 mg cm^{-2})	CuHCF<br>(5.0 mg cm^{-2})	16.5<br>(N/A)	74.3%, 1240 cycles, 2.0 A g^{-1}	[S27]
h-MoO_3<br>(2.0–3.0 mg cm^{-2})	CuHCF<br>(2.0–3.0 mg cm^{-2})	21.3<br>(N/A)	92.4%, 2000 cycles, 1.0 A g^{-1}	[S28]

Note: N/P ratio denotes the negative-to-positive capacity ratio.

Section S3. Supplementary References

[S1] Z. Tian, V. S. Kale, S. Thomas, S. Kandambeth, I. Nadinov, Y. Wang, W. Wahyudi, Y. Lei, A.-H. Emwas, M. Bonneau, O. Shekhah, O. M. Bakr, O. F. Mohammed, M. Eddaoudi, H. N. Alshareef, *Adv. Mater.* **2024**, *36*, 2409354.

[S2] J. Liu, K. Guo, W. Guo, J. Chang, Y. Li, F. Bao, *Angew. Chem. Int. Ed.* **2025**, 64, e202424494.

[S3] Z. Tian, V. S. Kale, Y. Wang, S. Kandambeth, J. Czaban-Jozwiak, O. Shekhah, M. Eddaoudi, H. N. Alshareef, *J. Am. Chem. Soc.* **2021**, 143, 19178.

[S4] H. Fei, F. Yang, Z. Jusys, S. Passerini, A. Varzi, *Adv. Funct. Mater.* **2024**, 34, 2404560.

[S5] H. Lu, J. Hu, K. Zhang, Y. Zhang, B. Jiang, M. Zhang, S. Deng, J. Zhao, H. Pang, B. Xu, *Adv. Mater.* 2024, 36, 2408396.

[S6] P. Liu, Z. Song, Q. Huang, L. Miao, Y. Lv, L. Gan and M. Liu, *Energy Environ. Sci.*, 2025, **18**, 5397-5406.

[S7] M. Gao, Z. Wang, Z. Liu, Y. Huang, F. Wang, M. Wang, S. Yang, J. Li, J. Liu, H. Qi, P. Zhang, X. Lu, X. Feng, *Adv. Mater.* **2023**, *35*, 2305575.

[S8] S. Yang, W. Zhao, Y. Mi, B. Li, Y. Dong, K. Xie, W. Zhao, G. Long, P. Du, *Angew. Chem. Int. Ed.*. **2025**, *64*, e202511826.

[S9] X. Shi, H. Liu, D. Xu, Y. Yu, X. Lu, *J. Phys. Chem. C* **2023**, *127*, 6233-6238.

[S10] J. Han, M. Zarrabeitia, A. Mariani, M. Kuenzel, A. Mullaliu, A. Varzi, S. Passerini, *Adv. Mater.* **2022**, *34*, 2201877.

[S11] F. Ye, R. Pang, C. Lu, Q. Liu, Y. Wu, R. Ma, L. Hu, *Angew. Chem. Int. Ed.* **2023**, *62*, e202303480.

[S12] S. Zhang, K. Zhu, Y. Gao, D. Cao, *ACS Energy Lett.* **2023**, *8*, 889.

[S13] H. Zhang, Y. Tian, W. Wang, Z. Jian, W. Chen, *Angew. Chem. Int. Ed.* **2022**, *61*, e202204351.

[S14] Q. Liu, F. Ye, K. Guan, Y. Yang, H. Dong, Y. Wu, Z. Tang, L. Hu, *Adv. Energy Mater.* **2023**, *13*, 2202908.

[S15] H. Li, J. Yang, J. Cheng, T. He, B. Wang, *Nano Energy* **2020**, *68*, 104369.

[S16] L. Chen, W. Sun, K. Xu, Q. Dong, L. Zheng, J. Wang, D. Lu, Y. Shen, J. Zhang, F. Fu, H. Kong, J. Qin, H. Chen, *ACS Energy Lett.* **2022**, *7*, 1672.

[S17] X. Wu, Y. Qi, J. J. Hong, Z. Li, A. S. Hernandez, X. Ji, *Angew. Chem. Int. Ed.* **2017**, *56*,

13026.

[S18] Z. Tian, J. Yin, T. Guo, Z. Zhao, Y. Zhu, Y. Wang, J. Yin, Y. Zou, Y. Lei, J. Ming, O. Bakr, O. F. Mohammed, H. N. Alshareef, *Angew. Chem. Int. Ed.* **2022**, *61*, e202213757.

[S19] L. Du, S. Bi, M. Yang, Z. Tie, M. Zhang, Z. Niu, *Proc. Natl. Acad. Sci.* **2022**, *119*, e2214545119.

[S20] H. Hong, J. Zhu, Y. Wang, Z. Wei, X. Guo, S. Yang, R. Zhang, H. Cui, Q. Li, D. Zhang, C. Zhi, *Adv. Mater.* **2024**, *36*, 2308210.

[S21] J. Liu, K. Wang, Y. Sun, H. Li, X. Han, X. Duan, Z. Huang, T. Ma, Nano Energy, **2025**, *136*, 110764.

[S22] P. Yi, Z. Li, L. Ma, B. Feng, Z. Liu, Y. Liu, W. Lu, S. Cao, H. Fang, M. Ye, J. Shen, *Adv. Mater.* **2024**, *36*, 2414379.

[S23] J. Dai, C. Yang, Y. Xu, X. Wang, S. Yang, D. Li, L. Luo, L. Xia, J. Li, X. Qi, A. Cabot, L. Dai, *Adv. Mater.* **2023**, *35*, 2303732.

[S24] C. Strietzel, M. Sterby, H. Huang, M. Strømme, R. Emanuelsson, M. Sjödin, *Angew. Chem. Int. Ed.* **2020**, *59*, 9631.

[S25] M. Zhu, L. Zhao, Q. Ran, Y. Zhang, R. Peng, G. Lu, X. Jia, D. Chao, C. Wang, *Adv. Sci.* 2022, *9*, 2103896.

[S26] L.Yan, Y. Qi, X. Dong, Y. Wang, Y. Xia, eScience, **2021**, *1*, 212.

[S27] S. Farai Kuchena, Y. Wang, *Chem. -Eur. J.* **2021**, *27*, 15450.

[S28] G. Liang, Y. Wang, Z. Huang, F. Mo, X. Li, Q. Yang, D. Wang, H. Li, S. Chen, C. Zhi, *Adv. Mater.* **2020**, *32*, 1907802.
