# Supplementary material for: Multi-electron nitrobenzothiadiazole sp-conjugated-alkynyl covalent organic frameworks for ammonium-ion batteries
Source: Nat Commun. 2026 Mar 7;17:3599. doi: 10.1038/s41467-026-70370-x (PMC13096558; doi:10.1038/s41467-026-70370-x)
Supplement: Supplementary file 3 — Description of Additional Supplementary Files [file 41467_2026_70370_MOESM3_ESM.pdf]

Description of Additional Supplementary Files

Supplementary Data 1. Monomer: Optimized molecular geometries of BTH, nitro-BTH, and TEB.

Supplementary Data 2. Adsorption energy: Geometrically optimized configurations of NH_4^+ at nitro/thiazole sites in the nitro-BTH framework.

Supplementary Data 3. Raction pathway: Geometrically optimized structures of nitro-BTH-COF with various NH_4^+ coordination modes.

Supplementary Data 4. Dynamics simulation: MD simulation snapshots showing the initial and final configurations of BTH-COF and nitro-BTH-COF.
